# Effects of dietary incorporation of *Radix rehmanniae praeparata* polysaccharide on growth performance, digestive physiology, blood metabolites, meat quality, and tibia characteristics in broiler chickens

**DOI:** 10.1016/j.psj.2023.103150

**Published:** 2023-09-24

**Authors:** Bing Yang, Xiaofeng Li, Amal MM. Baran, Abdel-Moneim Eid Abdel-Moneim

**Affiliations:** ⁎ College of Animal Science, Anhui Science and Technology University, Fengyang 233100, China; † Longyan University & Fujian Provincial Key Laboratory for Prevention and Control of Animal Infectious Diseases and Biotechnology, Longyan University, Longyan 364012, China; ‡ Poultry Breeding Department, Agricultural Research Center, Animal Production Research Institute, Egypt; § Biological Applications Department, Nuclear Research Center, Egyptian Atomic Energy Authority, Cairo 13759, Egypt

**Keywords:** *Radix rehmanniae preparata* polysaccharide, growth performance, digestive physiology, meat quality, broiler

## Abstract

*Radix rehmanniae preparata* polysaccharide (**RRPP**) is recognized as the primary bioactive compound in *Radix rehmanniae preparata* and has been extensively utilized in traditional Chinese medicine and functional food due to its diverse biological activities. However, this study has yet to explore the application of RRPP as a feed additive in broilers. This study investigated the effects of dietary RRPP on growth performance, meat quality, and physiological responses of broiler chickens. Two hundred eighty-eight 1-day-old Cobb 500 male broilers were randomly assigned to the 4 experimental groups with 6 replications and 12 birds/replicate. The 4 groups were fed the basal diet supplemented with 4 concentrations of RRPP (0, 300, 600, and 900 mg/kg, respectively). All RRPP levels did not affect the growth performance of broilers during the starter period (1–21 d), while during the grower (22–35 d) and overall (1–35 d) periods, body weight gain, feed conversion ratio, and European production efficiency index were linearly improved (*P* < 0.05) by incorporating RRPP at 600 and 900 mg/kg. Carcass characteristics, relative weight and length of intestinal segments, and meat quality and tibia criteria were not affected by dietary incorporation of RRPP. Dietary RRPP led to a linear increase (*P* < 0.05) in serum alkaline phosphatase, potassium, calcium and sulfhydryl levels, while reducing concentrations of hydrogen peroxide, LDL, triglycerides and total cholesterol. The addition of RRPP decreased (*P* < 0.05) the pH of the ileum and cecum at 21 and 35 d of age while not changing in the remaining intestinal segments. Dietary RRPP at 600 and 900 mg/kg linearly and quadratically (*P* < 0.05) increased the tibia ash content in chicken at 21 and 35 d of age. In conclusion, dietary supplementation of RRPP improved broiler chicken's growth, gut physiology, and tibia ash content, particularly at 600 and 900 mg/kg.

## INTRODUCTION

Modern intensive poultry production has achieved remarkable advancements in terms of productivity. However, antibiotic growth promoters (**AGPs**), mainly antibiotics and chemotherapeutics added to poultry feed to improve productivity and prevent diseases, have had detrimental effects on food safety and human health (Castanon, 2007; Abdel-Moneim et al., 2021). Issues such as drug residue and bacterial resistance have been noted as severe consequences of this practice (Abd El-Hack et al., 2020, Abd El-Hack et al., 2022). As a result, the poultry industry is poised to continue evolving toward more sustainable and responsible practices, meeting consumer demand for safe, healthy, and environmentally friendly products (Abd El-Hack et al., 2019). The switch toward replacing AGPs with novel alternatives has been driven by concerns over antibiotic resistance, as well as consumer demand for antibiotic-free poultry products. This trend has been observed globally, with the European Union banning the use of AGPs in animal feed in 2006 and other countries following suit (Castanon, 2007; Abd El-Moneim et al., 2020).

Alternative substances, including probiotics (Abdel-Moneim et al., 2020a, 2020c; Shehata et al., 2021), prebiotics (Abd El-Hack et al., 2022; Shehata et al., 2022), herbal extracts (Abd El-Hack et al., 2019; Elbaz et al., 2021; Mesalam et al., 2021), traditional Chinese medicine (**TCM**) (Dosoky et al., 2021; Saleh et al., 2021b; Wang et al., 2022), essential oils (Abd El-Hack et al., 2020; Elbaz et al., 2022), enzymes (Saleh et al., 2020, 2021a, 2022), and improved management practices (Abo Ghanima et al., 2020; Saleh et al., 2021c), can enhance the health and productivity of birds without the need for AGPs. Among these, herbal extracts and TCM have emerged as promising alternatives due to their natural plant products that possess antimicrobial, antioxidant, and immunomodulatory properties. Using Chinese herbal medicine in poultry aligns with the criteria for disease prevention in developing green products. Recent studies have highlighted the benefits of plant-derived bioactive compounds, such as polysaccharides, for enhancing growth performance, carcass traits, and meat quality in poultry production (Wang et al., 2022). Polysaccharides are practical components of medicinal plants that have been widely studied, and plant extracts containing natural polysaccharides have gained attention as an alternative to antibiotic additives due to their lower toxicity, multiple functions, and minimal side effects (Shan et al., 2019; Abdel-Moneim et al., 2020b).

*Radix rehmanniae praeparata* (**RRP**) is a TCM herb that contains active ingredients such as iridoid glycosides and polysaccharides, which have been shown to have antimicrobial and anti-inflammatory properties (Zhang et al., 2008). *Radix rehmanniae praeparata* polysaccharides (**RRPP**) are among the major active chemical components of RRP (Liang et al., 2013; Liu et al., 2017), and several studies have reported that they possess various pharmacological effects in rats and human cells, such as antioxidant properties (Fu et al., 2019; Zhou et al., 2020), anticancer effects (Xu et al., 2017; Li et al., 2018), immunomodulatory activities (Wang et al., 2018), and anti-inflammatory properties (Zhang et al., 2008). However, most studies on RRP have focused on its beneficial effects on humans as traditional Chinese medicine, with limited research on its potential use in poultry production.

To the best of our knowledge, the effect of dietary supplementation of RRPP in broilers has yet to be evaluated. Hence, the current study aimed to investigate the effects of RRPP supplementation on growth performance, blood metabolites, digestive physiology, meat quality, and tibia characteristics in broiler chickens.

## MATERIALS AND METHODS

### Ethics Statement

All study procedures were reviewed and approved by the Institutional Animal Care and Use Committee of Anhui Science and Technology University, Fengyang, Anhui Province in China (ECASTU-2015-P08).

### RRPP Composition Analysis

RRPP was provided by Shaanxi Hannah Biotechnology Co., Ltd. (Xi'an, China). The polysaccharides content in RRPP was assessed using the phenol-sulfuric acid method. The percentages of crude protein, ether extract, neutral detergent fiber, acid detergent fiber, and crude ash in RRPP were determined following the method described by da Teixeira et al. (2018).

### Experimental Design and Bird Management

A total of 288 one-day-old male Cobb 500 broiler chicks (45.11 ± 0.99 g) provided by the broiler hatchery of Bengbu Dacheng Food Co., Ltd. (Anhui, China) were randomly allocated to 4 treatment groups, with 6 replicates of 12 birds each. The experimental groups were fed a mash corn-soybean meal basal diet without or with RRPP supplementation at 0, 300, 600, and 900 mg/kg, respectively. The ingredient composition, estimated nutrient content, and determined chemical values of the basal diets are listed in Table 1. The trial lasted 35 d, divided into starter (1–21 d) and grower (22–35 d) periods. Birds were raised in floor cages under controlled environmental conditions, with an 23L:1D lighting regime and ad libitum access to water and mashed diets throughout the experimental period. Room temperature was maintained at 35°C for the first 3 d and gradually decreased to 20°C until the end of the experiment.

**Table 1:** Ingredient and composition of the basal diet.

Items	Starter (1–21 d)		Grower (22–35 d)
Ingredients (g/kg)			
Corn	514.2		566.6
Corn starch	10.0		10.0
Soybean meal	386.5		341.0
Fish meal	35.0		20.0
Soybean oil	30.0		35.0
Dicalcium phosphate	9.5		11.0
Ground limestone	9.0		10.5
Iodine salt	2.5		2.5
DL-methionine	0.9		1.0
Vitamin-mineral premix^1^	2.4		2.4
Nutrient content^2^			
Crude protein	231.2		204.7
AME (MJ/kg)	11.35		12.46
Methionine	4.8		4.3
Lysine	12.5		11.4
Methionine + cysteine	8.4		7.6
Nonphytate phosphorus	4.2		3.7
Calcium	10.3		8.8

1 Vitamin-mineral premix provided per kg diet: IU: vit. A 4,000,000, vit. D_3_ 500,000; g: vit. E 16.7, vit. K 0.67, vit. B_1_ 0.67, vit. B_2_ 2, vit. B_6_ 67, vit. B_12_ 0.004, nicotinic acid 16.7, pantothenic acid 6.67, biotin 0.07, folic acid 1.67, choline chloride 400, Zn 23.3, Mn 10, Fe 25, Cu 1.67, I 0.25, Se 0.033, Mg 133.4.

2 Calculated according to NRC (1994).

### Growth Performance

On d 21 and 35, birds were weighed, and feed intake (**FI**) was measured pen-based. The data were used to calculate the body weight gain (**BWG**), feed conversion ratio (**FCR**), and European production efficiency index (**EPEI**) for the experimental phases as described by Abdel-Moneim et al. (2022).

### Carcass Traits

On d 35, 6 chickens per group were randomly selected and manually slaughtered after 12-h fasting. The blood samples were collected for biochemical measurements. The hot carcass, pectoral and leg muscles, abdominal fat, claw, and wing were dissected and weighed to calculate the percentage of dressing, pectoral muscle, leg muscle, abdominal fat, claw, and wing, respectively.

### Serum Biochemical Analysis

Collected blood samples were centrifuged at 3,500 × *g* for 15 min at 4°C, and sera samples were obtained and stored at −80°C. Serum levels of albumin, alkaline phosphatase (**ALP**), alanine transaminase (**ALT**), triglyceride (**TG**), low-density lipoprotein (**LDL**), high-density lipoprotein (**HDL**), total cholesterol (**TC**), urea nitrogen (**UN**), creatinine, calcium, potassium, chlorine, hydrogen peroxide, and sulfhydryl were measured colorimetrically using the kits purchased from the Nanjing Jiancheng Institute of Bioengineering (Jiangsu, China).

### The Length of the Intestine

At d 21 and 35, 6 birds per treatment were randomly selected and weighed separately after 12 h feed withdrawal. After the broilers were manually slaughtered by severing the carotid artery and jugular vein, the segments of the duodenum, jejunum, ileum, and cecum were collected, and the length of each segment was measured with a tape. The jejunum and ileum are delimited by the yolk pedicle.

### Gastrointestinal pH and Relative Organ Weights

At d 21 and 35, the organs, including ingluvies, proventriculus, gizzard, duodenum, jejunum, ileum, and cecum, were dissected in the 2 phases, respectively. Then, a small incision was made in the middle of each segment, and immediately the electrode was utterly embedded into intestinal content. The pH value was determined using the pH meter (Shenzhen Jige Electromechanical Equipment Co., Ltd., Shenzhen, China). After removing chyme, the organs were weighed and the relative organ weights were calculated as a percentage of live body weight.

### Tibia Characteristics

The tibias were removed and cleaned of the adhering tissue to determine bone physical characteristics (e.g., the weight, length, width, and relative weight) and chemical composition (e.g., ash content) at d 21 and 35. The tibia ash content was evaluated with the method of Li et al. (2021). In brief, the fresh weight of the left tibia was determined. The tibias were degreased with petroleum ether for 96 h and dried at 105°C for 24 h. Then, the defatted tibias were ashed in a muffle furnace at 550°C for 12 h. Finally, the tibia ash was measured, and the content was calculated as the percentage of the fresh weight.

### Meat Quality

Meat color and pH were determined using the method described by Hou et al. (2020). In brief, the physical characteristics of each muscle sample were immediately assessed after slaughter. Meat color lightness (L*), redness (a*), and yellowness (b*) were measured using a CR-10 Plus chromameter (Zibo Diye Instrument Equipment Co., Ltd., Zibo, China). The pH was measured using the aforementioned pH meter. The electrodes were wholly embedded in the meat samples to make them entirely in contact with the tissue fluid in the muscle, and the pH value was recorded after the pH meter reading was stable.

Drip loss was estimated by the suspension method as the difference between the final and initial weights of a meat sample suspended in a self-sealing bag, stored at 4°C for 24 h, and expressed as the percentage of the initial weight. These procedures were performed in triplicate, and average values were calculated for analysis. In addition, cooking loss was measured using the method described by Honikel (1998). In brief, the fresh muscle slices (4 × 3 × 1 cm^3^) were weighed, placed in a plastic self-sealing bag, and cooked to an internal temperature of 70°C for 15 min in a water bath with a temperature of 80°C. Then water cooling blotted dry and weighed. The drip loss and cooking loss were calculated as (initial weight − final weight)/initial weight × 100%.

### Statistical Analysis

The differences among the groups were statistically analyzed by 1-way analysis of variance with SPSS software (version 21.0; SPSS Inc., Chicage IL USA). Shapiro-Wilk and Levene's tests were used to test the normal distribution of data as well as the homogeneity of variance. Tukey's multiple comparison test was used to determine the significance of mean differences, and the differences were indicated statistically significant at *P* < 0.05.

## RESULTS

### Composition of RRPP

Table 2 presents the composition of RRPP, showing the percentages of polysaccharide, crude protein, ether extract, neutral detergent fiber, acid detergent fiber, and crude ash as 50.18, 1.31, 8.52, 6.65, 1.20, and 2.02%, respectively.

**Table 2:** The composition of *Radix rehmanniae praeparata* polysaccharide.

Ingredients	Content (%)
Polysaccharide	50.18
Crude protein	1.31
Ether extract	8.52
Neutral detergent fiber	6.65
Acid detergent fiber	1.20
Crude ash	2.02

### Growth Performance

The impact of dietary RRPP inclusion on the growth performance of broiler chickens is outlined in Table 3. During the starter period (1–21 d), all RRPP levels showed no significant effect on growth performance criteria. However, in the grower (22–35 d) and overall (1–35 d) periods; BWG, FCR, and EPEI were linearly improved by incorporating RRPP at 600 and 900 mg/kg, while FI remained unaffected.

**Table 3:** Effect of dietary *Radix rehmanniae praeparata* polysaccharide (RRPP) on growth performance of broiler chickens from 1 to 35 d of age.

Parameter^1^	Dietary RRPP level, mg/kg				SEM^2^	*P* values		
	0	300	600	900		RRPP	Linear	Quadratic
1–21 d								
BWG, g/bird/d	35.88	35.08	36.65	36.32	0.547	0.729	0.584	0.840
FI, g/bird/d	46.46	46.68	46.56	46.25	0.095	0.480	0.417	0.191
FCR, g feed/g/gain	1.301	1.342	1.271	1.276	0.019	0.586	0.426	0.650
EPEI	295.2	281.8	305.8	302.6	8.469	0.787	0.572	0.781
22–35 d								
BWG, g/bird/d	64.10^c^	66.37^bc^	68.05^ab^	70.84^a^	0.775	0.006	0.001	0.828
FI, g/bird/d	128.5	127.3	128.5	129.5	0.315	0.101	0.124	0.076
FCR, g feed/g/gain	2.006^a^	1.925^ab^	1.891^ab^	1.829^b^	0.023	0.034	0.005	0.805
EPEI	604.2^c^	637.0^bc^	668.9^ab^	703.3^a^	10.93	0.002	>0.001	0.959
1–35 d								
BWG, g/bird/d	47.17^c^	47.60^bc^	49.21^ab^	50.13^a^	0.390	0.010	0.001	0.690
FI, g/bird/d	79.29	78.94	79.35	79.55	0.148	0.555	0.381	0.379
FCR, g feed/g/gain	1.682^a^	1.659^ab^	1.613^bc^	1.588^c^	0.012	0.006	0.001	0.959
EPEI	288.5^c^	294.8^bc^	313.4^ab^	324.1^a^	4.514	0.007	0.001	0.747

Means in the same row with different superscripts (a–c) are significantly different.

1 BWG = body weight gain; FI = feed intake; FCR = feed conversion ratio; EPEI = European production efficiency index.

2 SEM = standard error of means.

### Carcass Traits

Table 4 presents the effect of dietary RRPP supplementation on broiler carcass traits at 35 d of age. All studied RRPP levels had no impact on dressing percentage, ingluvies, proventriculus, gizzard, pectoral muscle, leg muscle, abdominal fat, claw, and wing.

**Table 4:** Effect of dietary *Radix rehmanniae praeparata* polysaccharide (RRPP) on carcass traits (%) of broiler chickens at 35 d of age.

Parameter	Dietary RRPP level, mg/kg				SEM^1^	*P* values		
	0	300	600	900		RRPP	Linear	Quadratic
Dressing	74.32	74.59	73.95	74.69	0.267	0.791	0.853	0.679
Ingluvies	0.247	0.292	0.291	0.316	0.008	0.101	0.355	0.410
Proventriculus	0.420	0.421	0.430	0.423	0.004	0.836	0.662	0.627
Gizzard	1.699	1.627	1.650	1.698	0.019	0.480	0.914	0.141
Pectoral muscle	19.16	20.10	19.24	19.15	0.259	0.528	0.705	0.344
Leg muscle	15.02	15.17	15.18	15.46	0.223	0.931	0.549	0.891
Abdominal fat	0.993	1.059	0.946	0.968	0.034	0.703	0.562	0.760
Claw	4.063	4.265	4.219	4.142	0.083	0.858	0.812	0.440
Wing	8.067	8.011	7.858	7.678	0.080	0.328	0.078	0.697

Values provided represent means that have been deemed statistically significant at (*P* < 0.05).

1 SEM = standard error of means.

### Serum Biochemical Indices

The effects of including RRPP in broiler diets on their serum biochemical constituents are presented in Table 5. Dietary levels of RRPP did not affect serum levels of albumin, ALT, creatinine, HDL, and chloride compared to the control group. However, serum ALP, potassium and sulfhydryl concentrations showed a linear increase (*P* > 0.05) in birds fed RRPP at 900 mg/kg. The urea level exhibited a quadratic decrease (*P* > 0.01) in groups fed diets with 300 and 600 mg/kg RRPP. Hydrogen peroxide and LDL concentrations were reduced (*P* > 0.01) in all treated groups, while total cholesterol and triglycerides showed reductions in groups fed 900 mg/kg and 600 and 900 mg/kg RRPP, respectively, compared to the control. Calcium level demonstrated a linear elevation (*P* > 0.05) in the 600 and 900 mg/kg RRPP groups compared to the control.

**Table 5:** Effect of dietary *Radix rehmanniae praeparata* polysaccharide (RRPP) on serum biochemical constituents of broiler chickens at 35 d of age.

Parameter^1^	Dietary RRPP level, mg/kg				SEM^2^	*P* values		
	0	300	600	900		RRPP	Linear	Quadratic
Albumin, g/L	60.17	69.89	59.63	66.97	3.458	0.691	0.758	0.872
ALT, U/100 mL	0.208	0.209	0.210	0.208	0.001	0.766	0.862	0.340
ALP, U/100 mL	9.291^b^	9.165^b^	9.798^b^	11.77^a^	0.349	0.015	0.005	0.076
Urea, mg/L	6.509^a^	4.902^bc^	4.153^c^	5.848^ab^	0.281	0.005	0.162	0.001
Creatinine, mmol/L	83.52	82.89	84.63	86.59	7.601	0.912	0.884	0.938
Cholesterol, mmol/L	14.38^a^	13.09^a^	12.93^a^	10.91^b^	0.395	0.007	0.001	0.550
Triglycerides, mmol/L	10.38^b^	10.58^a^	9.801^c^	9.440^d^	0.108	>0.001	>0.001	0.001
LDL, mmol/L	6.384^a^	4.713^b^	4.687^b^	4.779^b^	0.205	0.001	0.001	0.004
HDL, mmol/L	5.922	6.261	6.281	4.241	0.324	0.065	0.065	0.052
Calcium, mmol/L	1.886^b^	2.311^ab^	2.954^a^	2.610^a^	0.106	0.037	0.007	0.270
Potassium, mmol/L	1.638^b^	1.938^ab^	1.911^ab^	2.288^a^	0.085	0.046	0.009	0.799
Chloride, mmol/L	63.75	60.92	61.64	63.29	1.006	0.754	0.945	0.306
Sulfhydryl, mmol/L	0.465^b^	0.597^ab^	0.587^ab^	0.694^a^	0.030	0.046	0.010	0.809
Hydrogen peroxide, mmol/L	217.3^a^	143.5^b^	147.0^b^	104.4^b^	12.96	0.005	0.001	0.417

Means in the same row with different superscripts (a–d) are significantly different

1 ALT = alanine transaminase; ALP = alkaline phosphatase; LDL = low-density lipoprotein cholesterol; HDL = high-density lipoprotein cholesterol.

2 SEM = standard error of means.

### Gut Morphology

Table 6 demonstrates the effects of dietary RRPP supplementation on gut morphology in broiler chickens at 21 and 35 d of age. RRPP inclusion did not affect the relative weight and length of the duodenum, jejunum, ileum, and cecum at both ages. However, at 35 d of age, the total intestinal weight linearly increased with 900 mg/kg RRPP inclusion compared to the other groups.

**Table 6:** Effect of dietary *Radix rehmanniae praeparata* polysaccharide (RRPP) on gut morphology of broiler chickens at 21 and 35 d of age.

Parameter	Dietary RRPP level, mg/kg				SEM^1^	*P* values		
	0	300	600	900		RRPP	Linear	Quadratic
21 d								
The relative weight of intestinal fragments, %								
Duodenum	18.13	18.00	19.23	18.59	0.430	0.101	0.143	0.080
Jejunum	38.91	37.52	38.90	39.15	0.489	0.667	0.651	0.434
Ileum	33.48	32.56	29.44	29.90	0.763	0.165	0.045	0.635
Cecum	9.476	11.92	10.43	12.36	0.470	0.099	0.077	0.768
Total, g	34.26	33.62	34.34	34.76	0.739	0.966	0.760	0.741
The relative length of intestinal fragments, %								
Duodenum	14.70	14.18	14.78	15.13	0.275	0.707	0.468	0.461
Jejunum	36.51	36.08	36.48	36.13	0.248	0.909	0.752	0.938
Ileum	36.62	37.43	36.91	36.61	0.387	0.887	0.885	0.517
Cecum	12.17	12.31	11.83	12.13	0.216	0.903	0.774	0.877
Total, cm	153.6	153.4	154.9	154.6	1.068	0.964	0.688	0.979
Density	0.223	0.219	0.222	0.225	0.005	0.983	0.870	0.726
35 d								
The relative weight of intestinal fragments, %								
Duodenum	17.38	17.59	16.68	17.00	0.225	0.532	0.335	0.909
Jejunum	37.15	37.57	39.43	38.62	0.398	0.168	0.078	0.422
Ileum	35.97	35.52	35.08	35.50	0.329	0.850	0.568	0.547
Cecum	9.503	9.320	8.802	8.878	0.155	0.323	0.097	0.676
Total, g	42.68^b^	42.76^b^	43.68^ab^	46.57^a^	0.627	0.083	0.023	0.226
The relative length of intestinal fragments, %								
Duodenum	13.88	14.32	13.78	13.80	0.202	0.793	0.693	0.634
Jejunum	34.35	33.57	34.60	34.00	0.269	0.599	0.992	0.880
Ileum	35.79	35.59	34.98	35.96	0.317	0.750	0.972	0.393
Cecum	15.99	16.52	16.64	16.25	0.257	0.835	0.717	0.409
Total, cm	182.4	184.1	184.2	185.0	1.303	0.930	0.542	0.872
Density	0.234	0.233	0.237	0.252	0.004	0.273	0.102	0.283

Means in the same row with different superscripts (a, b) are significantly different.

1 SEM = standard error of means; Density = total weight/total length ratio.

### pH of Digestive Organs

As indicated in Table 7, the addition of RRPP decreased (*P* < 0.05) the pH of the ileum and cecum at 21 and 35 d of age. However, RRPP supplementation had no significant effect on the pH of ingluvies, proventriculus, gizzard, duodenum, and jejunum at both ages.

**Table 7:** Effect of dietary *Radix rehmanniae praeparata* polysaccharide (RRPP) on digestive organs' pH of broiler chickens at 21 and 35 d of age.

Parameter	Dietary RRPP level, mg/kg				SEM^1^	*P* values		
	0	300	600	900		RRPP	Linear	Quadratic
21 d								
Ingluvies	6.302	6.174	6.146	6.320	0.063	0.723	0.965	0.270
Proventriculus	4.376	4.412	4.488	4.270	0.072	0.788	0.727	0.417
Gizzard	2.600	2.494	2.524	2.580	0.051	0.896	0.952	0.472
Duodenum	6.046	6.120	6.084	6.032	0.067	0.973	0.906	0.671
Jejunum	6.324	6.396	6.328	6.390	0.051	0.944	0.795	0.694
Ileum	6.170^a^	5.386^b^	5.598^b^	5.698^b^	0.083	0.001	0.027	0.001
Cecum	6.030^a^	5.536^b^	5.474^b^	5.430^b^	0.080	0.014	0.005	0.094
35 d								
Ingluvies	6.266	6.270	6.252	6.222	0.050	0.989	0.760	0.877
Proventriculus	4.184	4.450	4.226	4.416	0.085	0.643	0.599	0.833
Gizzard	3.048	3.042	3.260	2.950	0.075	0.546	0.913	0.335
Duodenum	6.008	5.976	6.034	6.024	0.035	0.949	0.754	0.884
Jejunum	6.394	6.330	6.320	6.328	0.048	0.952	0.657	0.730
Ileum	6.258^a^	5.676^b^	5.688^b^	5.346^b^	0.100	0.003	0.001	0.412
Cecum	6.052^a^	5.192^b^	5.202^b^	5.238^b^	0.089	<0.001	<0.001	<0.001

Means in the same row with different superscripts (a, b) are significantly different.

1 SEM = standard error of means.

### Meat Quality

Table 8 shows the effect of dietary RRPP on meat quality traits in broiler chickens at 35 d of age. RRPP addition had no impact on the color, pH_45_, pH_24_, drip loss, and cooking loss of the pectoral and leg muscles.

**Table 8:** Effect of dietary *Radix rehmanniae praeparata* polysaccharide (RRPP) on meat quality traits of broiler chickens at 35 d of age.

Parameter	Dietary RRPP level, mg/kg				SEM^1^	*P* values		
	0	300	600	900		RRPP	Linear	Quadratic
Pectoral muscle								
L_45 min_	52.48	52.31	52.42	52.36	0.074	0.875	0.717	0.724
a_45 min_	11.56	11.39	11.71	11.64	0.071	0.447	0.377	0.725
b_45 min_	7.553	7.779	7.553	7.453	0.133	0.868	0.682	0.573
pH_45 min_	7.126	6.606	6.788	6.810	0.107	0.361	0.466	0.232
pH_24 h_	6.306	6.232	6.106	6.370	0.048	0.285	0.885	0.097
Drip loss_24 h_/%	4.650	4.666	4.568	4.612	0.049	0.910	0.652	0.892
Cooking loss/%	23.01	22.71	21.45	22.67	0.343	0.414	0.466	0.286
Leg muscle								
L_45 min_	52.83	52.13	52.70	52.34	0.198	0.610	0.627	0.686
a_45 min_	11.57	11.03	11.48	11.71	0.157	0.487	0.555	0.243
b_45 min_	7.910	7.795	7.549	7.377	0.152	0.750	0.295	0.947
pH_45 min_	7.108	7.002	7.172	7.510	0.092	0.242	0.101	0.227
pH_24 h_	7.108	7.002	7.172	7.503	0.099	0.453	0.562	0.895
Drip loss_24 h_/%	4.648	4.766	4.566	4.600	0.049	0.907	0.645	0.902
Cooking loss/%	23.09	22.91	21.94	22.76	0.343	0.364	0.597	0.234

Means in the same row with different superscripts are significantly different.

1 SEM = standard error of means.

### Tibia Traits

As depicted in Table 9, RRPP supplementation had no significant effects on the tibia's length, width, weight, and relative weight at 21 and 35 d of age. Interestingly, RRPP at the levels of 600 and 900 mg/kg linearly and quadratically increased (*P* < 0.05) the tibia ash content in chicken at 21 d of age. RRPP at 300, 600, and 900 mg/kg linearly and quadratically increased (*P* < 0.05) tibia ash content in chicken at 35 d of age.

**Table 9:** Effect of dietary *radix Rehmanniae praeparata* polysaccharide (RRPP) on tibia characteristics of broiler chickens at 21 and 35 d of age.

Parameter	Dietary RRPP level, mg/kg				SEM^1^	*P* values		
	0	300	600	900		RRPP	Linear	Quadratic
21 d								
Tibia length, cm	4.448	4.108	4.494	4.618	0.122	0.525	0.431	0.363
Tibia width, cm	5.918	6.068	5.692	5.960	0.121	0.764	0.829	0.819
Tibia ash content, %	20.96^c^	22.99^bc^	31.18^a^	24.73^b^	0.958	<0.001	<0.001	<0.001
Tibia weight, g	6.793	6.325	6.741	6.374	0.084	0.084	0.225	0.740
Tibia relative weight, %	0.859	0.818	0.829	0.791	0.017	0.568	0.216	0.967
35 d								
Tibia length, cm	8.692	8.522	8.584	8.556	0.073	0.879	0.626	0.654
Tibia width, cm	8.014	7.806	8.046	8.472	0.150	0.487	0.251	0.311
Tibia ash content, %	36.09^b^	37.55^a^	38.62^a^	38.11^a^	0.282	0.002	0.001	0.023
Tibia weight, g	10.12	9.696	9.678	10.06	0.230	0.873	0.929	0.420
Tibia relative weight, %	0.576	0.564	0.570	0.562	0.012	0.982	0.767	0.941

Means in the same row with different superscripts (a–c) are significantly different.

1 SEM = standard error of means.

## DISCUSSION

The utilization of phytogenic feed additives and their derivatives to improve the health conditions of broilers has gained popularity over time (Abdel-Moneim et al., 2020b). Among these additives, polysaccharides have been recognized for their diverse bioactivities, encompassing enhancements in immunity, antioxidant properties, anticancer potential, antiobesity effects, and hypoglycemic benefits. Numerous studies have investigated the bioactive effects of plant-derived polysaccharides, such as those from *Camellia oleifera, Astragalus membranaceus, Ficus carica, Lycium barbarum*, and *Acanthopanax senticosus*. Furthermore, Liu et al. (2021) reported that *Yingshan yunwu* tea-polysaccharides as feed additives can improve immune status, carcass traits, meat quality, gut health, and microbiota in broilers. Nevertheless, to the best of our knowledge, no previous studies investigated the effects of dietary RRPP supplementation on broiler chickens. The selection of the specific dosage levels in this study was based on those used in previous research examining the impacts of polysaccharides from various plants. In this study, RRPP supplementation at levels of 600 and 900 mg/kg improved BWG, FCR, and EPEI at growing and overall periods, while carcass traits were not altered. These results are align with those found by Long et al. (2020), who reported that *Lycium barbarum* polysaccharides could improve the ADWG of Arbor Acres broilers. It was also consistent with the previous report of Wu (2018), who reported that the polysaccharides from *Astragalus membranaceus* increased growth performance of broilers. The favorable impact of RRPP on broiler growth may be attributed to their ability to induce the mRNA expression of digestive enzymes (Long et al., 2020). This, in turn, leads to an increase in the activities of protease, amylase, and lipase, thereby contributing to improved digestive function. Another potential mechanism underlying the beneficial effects of RRPP on broiler growth performance is its ability to enhance nutrient absorption in the intestine. Ren et al. (2017) demonstrated that *L. barbarum* polysaccharides improved intestinal permeability and histology in rats.

In this study, the inclusion of RRPP led to an increase in the serum levels of ALP, Ca, and K. Alkaline phosphatase functions as a crucial enzyme involved in osteoid formation and mineralization. In individuals with normal liver function, elevated ALP levels serve as a nonspecific marker indicating heightened bone formation and increased osteoblast activity (Kumar and Maurya, 2018). This suggests that RRPP supplementation could stimulate bone growth and mineralization, consequently resulting in elevated ALP levels. The process of bone formation necessitates essential minerals such as calcium and potassium (Palacios, 2006). The heightened levels of these minerals in the bloodstream of birds indicate that the addition of RRPP might enhance the absorption of these minerals from the digestive system, subsequently leading to improved utilization within the body.

The hypocholesterolemic and hypolipidemic potential of RRPP is greatly noticed in the present study, as serum cholesterol, triglycerides, and LDL were reduced by the dietary inclusion of RRPP. Plant-derived polysaccharides exert diverse effects as cholesterol-lowering agents, influenced by factors like their physicochemical properties, sugar composition, and gut microbiota fermentation (Huang et al., 2018; Naumann et al., 2019; Marasca et al., 2020; Silva et al., 2021). These effects include bile salt sequestration and inhibition of cholesterol esterase in the intestinal lumen, impacting cholesterol bioavailability (Hui and Howles, 2005). Viscosity changes in intestinal digesta caused by polysaccharides impede cholesterol-loaded micelles diffusion to the intestinal epithelium, limiting absorption (Naumann et al., 2019). Furthermore, bile salt sequestration by polysaccharides reduces cholesterol emulsification, enhancing fecal excretion (Pengzhan et al., 2003). Reduced bile salt recirculation prompts higher endogenous cholesterol conversion to primary bile salts (Garcia-Diez et al., 1996). Additionally, microbiota fermentation of polysaccharides produces short-chain fatty acids (**SCFA**), hindering cholesterol synthesis, and promoting secondary bile salt formation, which is crucial for emulsification (Wahlström et al., 2016). The interplay of these mechanisms and their potential synergy can explain the hypocholesterolemic impact of RRPP presented in this study.

The findings of this study demonstrated the antioxidant-promoting abilities of RRPP, as evidenced by determining serum hydrogen peroxide and sulfhydryl levels. Hydrogen peroxide (**H_2_O_2_**), generated by inflammatory and vascular cells, initiates oxidative stress by engendering OH and O_2_ radicals through the Fenton's reaction with Fe^2+^ (Ransy et al., 2020) and activating NADPH oxidase (Coyle et al., 2006). Conversely, sulfhydryl groups of thiols act as critical agents in counteracting the reactive oxygen species (**ROS**) generated during heightened oxidative stress (Erkus et al., 2015). The plasma thiol pool primarily comprises protein thiols like albumin alongside low molecular mass thiols such as glutathione, gamma glutamine, cysteine, and homocysteine (Turell et al., 2013). Recently, thiol level and thiol/disulfide homeostasis have been considered novel oxidative stress markers (Kundi et al., 2015; Altıparmak et al., 2016). Remarkably, this study demonstrates that RRPP supplementation elevates serum sulfhydryl levels while concurrently reducing H_2_O_2_ concentration, indicative of RRPP's robust ROS scavenging capabilities. These results resonate with Long et al. (2020), who observed similar trends in *Lycium barbarum* polysaccharide treatment, enhancing antioxidant parameters in the serum and liver of Arbor Acres broilers. This enhancement was reflected in augmented glutathione peroxidase and superoxide dismutase activities and decreased malondialdehyde levels. Similar outcomes were observed in vitro by Liu et al. (2023) and in vivo by Fu et al. (2019) and Zhou et al. (2020) in rat models.

The intestine serves as the primary site for nutrient digestion and absorption, playing a crucial role in overall immune function and animal health. Alterations in the length of the small intestine can impact nutrient uptake. In this investigation, no significant differences were observed in the lengths of the duodenum, jejunum, ileum, and cecum between the treated and untreated groups. Notably, this study assessed the influence of RRPP supplementation on the pH levels within each intestinal segment. While RRPP utilization did not result in pH changes in the segments before the ileum, a notable decrease in pH was observed in the ileum and cecum compared to control birds. Furthermore, the addition of RRPP at 900 mg/kg conspicuously elevated the relative weight of the jejunum in broilers. *Radix rehmanniae praeparata* polysaccharides can influence the reduction of intestinal pH in birds through several mechanisms. These polysaccharides are often fermented in the ileum and cecum by the gut microbiota, leading to the production of SCFA, particularly acetate, propionate, and butyrate (Wahlström et al., 2016). These SCFA have the capacity to lower the pH of the intestinal environment as a result of their metabolism. The increase in SCFA levels leads to an accumulation of organic acids in the gut, thereby reducing the pH (Nogal et al., 2021). Additionally, the microbial fermentation of polysaccharides produces other metabolites, such as lactate and succinate, which further contribute to the acidic environment (Wassie et al., 2021; Wang et al., 2022). This decrease in intestinal pH can impact diverse physiological processes within birds, encompassing nutrient absorption, microbial populations, and overall gut health. This factor could offer an additional rationale for the observed enhancement in growth performance, as demonstrated in this study.

Over the past decades, enhancing the quality of raw meat for consumers has been a prominent focus. In this context, the impact of RRPP on broiler meat quality traits at 35 d of age was investigated. Notably, meat color values, pH levels, and water retention capacity are key nutritional indicators reflecting chicken meat quality. Therefore, pH_45 min_, pH_24 h_, L*, a*, and b* values drip loss, and cooking loss of breast and thigh meat were assessed. Supplementation of RRPP did not influence meat quality traits, including drip loss, pH, color, and cooking loss of breast and leg muscles, aligning well with a previous report (Wang et al., 2020). Nevertheless, our results diverge from another study indicating YingshanYunwu tea polysaccharides reduced breast muscle attributes and altered color and pH in Chongren Chickens (Zhao et al., 2020). Such variations could stem from differences in chicken breeds and mixture compositions.

Leg diseases, a prominent contributor to losses in broiler production, can lead to bone deformities, impaired coordination, and compromised animal welfare (Huang et al., 2019). A notable example is tibial dyschondroplasia, a common leg issue in broiler chickens that negatively impacts the quality of pectoral and leg muscles (Cao et al., 2020). In the current study, the addition of RRPP resulted in an increased tibia ash content while having no effect on tibia length, width, or relative weight in broiler chickens. This finding aligns with a report indicating that *Trametes versicolor* polysaccharides improved bone health in diabetic rats (Chen et al., 2015). The elevated ash content in the tibia reflects higher mineral content, including calcium and potassium. In this study, RRPP supplementation was shown to enhance mineral absorption, as evidenced by increased serum calcium and potassium levels. This heightened mineral uptake likely contributes to their deposition in bone tissues, ultimately leading to higher ash content in the tibia and improved bone mineralization. The intricate relationship between polysaccharide supplementation, mineral absorption, and bone health underscores the potential beneficial effects of these dietary additives on avian skeletal development.

As far as our knowledge extends, this study provides pioneering evidence that RRPP holds promise as a valuable feed additive for augmenting broiler performance and health. The incorporation of RRPP exhibited robust antioxidant and hypocholesterolemic effects, leading to improved growth performance and enhanced bone mineralization. The suggested inclusion levels of 600 and 900 mg/kg yielded encouraging outcomes. Nevertheless, further investigations are required to fully elucidate the underlying mechanisms and fine-tune RRPP incorporation levels for optimal outcomes in broiler production.
